# ASSOCIATIONS BETWEEN LONGITUDINAL LONELINESS, DNA METHYLATION AGE ACCELERATION, AND COGNITIVE FUNCTIONING

**DOI:** 10.1093/geroni/igae098.1353

**Published:** 2024-12-31

**Authors:** Morgan Lynch, Christopher Beam, Thalida Em Arpawong

**Affiliations:** University of Southern California, Los Angeles, California, United States; University of Southern California, Los Angeles, California, United States; University of Southern California, Los Angeles, California, United States

## Abstract

Loneliness may influence aging biomarkers related to cognitive functioning, for example, through accelerated DNA methylation (DNAm) aging. In a study involving 1,814 participants from the Health and Retirement Study, we investigated the role of six DNAm age acceleration measures (DNAmAA) in mediating the effects of baseline loneliness and latent longitudinal loneliness trajectories on cognitive abilities. Higher baseline loneliness and a moderate-intercept loneliness trajectory predicted poorer general cognitive ability and memory scores. Second-generation DNAmAA measures (specifically, PhenoAge, GrimAge, and DunedinPoAm38) mediated the effect of loneliness on overall cognitive ability and memory. Additionally, immediate and delayed memory scores were mediated by GrimAge Accel in the lowest and two highest latent loneliness trajectories. Notably, self-rated health, depressive symptoms, social isolation, and body mass index contributed significantly to total and mediated effects. The study suggests that DNAmAA, especially GrimAge, have promise for explaining the prospective association between loneliness and poorer cognitive functioning.

